# MULTILEVEL SELECTION WITH KIN AND NON-KIN GROUPS, EXPERIMENTAL RESULTS WITH JAPANESE QUAIL (*COTURNIX JAPONICA*)

**DOI:** 10.1111/evo.12062

**Published:** 2013-02-18

**Authors:** William M Muir, P Bijma, A Schinckel

**Affiliations:** 1 Purdue UniversityWest Lafayette, Indiana, 47906; 3 ABGC, Wageningen University6700 AH, Wageningen, The Netherlands

**Keywords:** Behavior, competition, selection—group/kin, quantitative genetics

## Abstract

An experiment was conducted comparing multilevel selection in Japanese quail for 43 days weight and survival with birds housed in either kin (K) or random (R) groups. Multilevel selection significantly reduced mortality (6.6% K vs. 8.5% R) and increased weight (1.30 g/MG K vs. 0.13 g/MG R) resulting in response an order of magnitude greater with Kin than Random. Thus, multilevel selection was effective in reducing detrimental social interactions, which contributed to improved weight gain. The observed rates of response did not differ significantly from expected, demonstrating that current theory is adequate to explain multilevel selection response. Based on estimated genetic parameters, group selection would always be superior to any other combination of multilevel selection. Further, near optimal results could be attained using multilevel selection if 20% of the weight was on the group component regardless of group composition. Thus, in nature the conditions for multilevel selection to be effective in bringing about social change maybe common. In terms of a sustainability of breeding programs, multilevel selection is easy to implement and is expected to give near optimal responses with reduced rates of inbreeding as compared to group selection, the only requirement is that animals be housed in kin groups.

The primary goal of most domestication programs is to maximize individual productivity or merit. However, breeding methods currently in common use assume individuals do not interact (Falconer and Mackay 1996; Lynch and Walsh 1998). Because social interactions are ubiquitous, further domestication, particularly to address animal wellbeing concerns, need to take such interactions into account (Craig and Muir 1998; Wade et al. 2010a). Consistent with these goals is the need to focus on the correct metric of productivity, which should be that of the herd, group, cage, or pen, not the individual. This is because high individual productivity may come at the expense of cage or pen mates due to negative social interactions, and as a result group productivity can be compromised. Moreover, social interactions can also result in injuries, stress, and mortalities, which in turn results in animal wellbeing concerns (Muir and Craig 1998; Muir and Cheng 2004). Hence, breeders need to develop selection strategies that lead to cooperative or even altruistic individuals, which may involve mechanisms proposed for the evolution of cooperation in natural populations, such as kin and group selection. In turn, results from breeders maybe of interest to evolutionary biologists because the effectiveness of those mechanisms can be evaluated empirically, which is difficult in natural populations.

Classical theory for evolution of social behavior is confusing and a topic of current debate (Wild et al. 2009; Okasha 2010; Wade et al. 2010b; Goodnight 2012). The usual examples of social evolution relate to altruism, where there is a fitness cost to the individual, such as with social insects, ants, bees, and termites, whereby an individual forfeits its ability to pass on genes in favor of a greater number of related offspring produced by kin. The dilemma with altruism is to explain how nature was able to achieve a positive response in fitness to negative selection, that is, selection against the individual in favor of its kin. Models to explain evolution of social behavior that has a fitness cost to the actor can be broadly separated into “kin selection” as opposed to “group selection” (Maynard-Smith 1964; Wade 1985; Goodnight 2005; Lehmann et al. 2007). Kin selection theory was developed by Hamilton (1963, 1964a,b) and later generalized by Price (1970, 1972a,b). Kin selection is based the concept of inclusive fitness (IF), that is, the sum of the effects of an allele on fitness of the individual possessing the allele (cost), and on all those it interacts with (benefit), weighted by relatedness between the focal individuals and the individuals receiving the benefit; IF = *rb* − *c*. The focus of kin selection theory is the individual, but in the context of those it interacts with. Individuals may or may not be organized into recognizable groups. Kin selection theory can explain the evolution of altruistic behavior, for example, when contributions of the individual to the fitness of the group (*b*) times the relationship of the individual to the group (*r*), is greater than the reproductive cost (*c*) to the individual (*rb* > *c*), known as Hamilton's rule.

Classical group selection was first proposed by Wynne-Edwards (1963), and expanded on by Williams (1966), as a mechanism for the evolution of cooperation among individuals of the same or different species, that is, stable ecosystems. Group selection was defined as reproduction or extinction of entire groups yielding group-level adaptations, that is, among group selection. Issues facing group selection as a mode of evolution include: between-group selection being opposed by individual selection within groups (Slatkin and Wade 1978; Wade et al. 2010b), emigration rate between groups, group size, degree of relationship among individuals within the group, and frequency with which entire groups become extinct (Goodnight and Stevens 1997; Wade et al. 1999; Goodnight 2005; Bijma and Wade 2008; Wade et al. 2010b). Group selection is most effective if groups exchange migrants rarely, group size is small, the relationships within a group are high, and entire groups become extinct, that is, no survivors from failed groups are allowed to mate in new groups. These conditions seem so rare that most consider classical group selection to be an improbable mechanism for evolution of cooperation, but it remains a current topic of debate (Lehmann et al. 2007; West et al. 2008; Wild et al. 2009; Wade et al. 2010b). Some contend that classical group selection can only be successful if counteracting effects of individual selection within groups is negligible, and contend such situation rarely, if ever, occur (Wild et al. 2009). The essential difference between the two evolutionary theories is the unit upon which selection acts, the individual or the group. Nevertheless, group selection has been shown to be very effective under a wide range of conditions (Goodnight and Stevens 1997). Examples include control of segregation distorter genes in mice (Lewontin 1962) and for population size in *Tribolium castaneum* (Wade 1976, 1980; Wade and McCauley 1980, 1984).

Kin and group selection theory usually focuses entirely on fitness, leaving phenotypic effects of alleles on trait values implicit (Gardner et al. 2011). In the kin-group selection debate, for example, so-called indirect genetic effects (IGEs) on trait values are widely ignored. Griffing (1967) termed the social, or competitive effects, as “associative effects,” which are now commonly referred to as IGEs (Agrawal et al. 2001; Bijma and Wade 2008; McGlothlin and Brodie 2009; Bijma 2010a,b; Wade et al. 2010b). An IGE is a heritable effect of an individual's genes on trait values of other individuals (Griffing, 1967; Moore et al., 1997). Theory and selection experiments have demonstrated that IGEs can substantially affect response to selection, such as reverse the direction of response (Griffing 1967; Craig and Muir 1996; Wolf et al. 1998; Muir 2005). Thus kin and group selection can explain the evolution of fitness cost and benefit of social interactions, but fall short in explaining response to selection in trait values affected by social interactions, such as those encountered in artificial breeding programs. In breeding programs the theoretical problem is how to achieve maximal response to selection as social interactions can cause individual selection to be suboptimal and even result in a negative response (Griffing 1967), for example, with cannibalism as observed in chickens (Craig and Muir 1996; Muir 1996), IGEs reversed the response so a negative response was observed to positive individual selection. We hypothesize that relatedness and group selection are needed as tools to re-reverse response, so that we get positive response to positive selection.

Muir and colleagues (Craig and Muir 1996; Muir 1996) directly compared individual versus group selection to reduce cannibalism and increase egg production in poultry (*Gallus gallus*) to alleviate the necessity to trim beaks to reduce stress and injury associated with pecking. Starting with the same base population, group level selection (half-sib families) for livability (days survival) and egg production dramatically improved survivability in a competitive colony cage environment, whereas individual selection not only failed, but resulted in a deterioration in livability. The realized heritability for group selection (response to selection relative to the selection differential of the parents) was initially greater than 1, which could be due to the reality that total heritable variation, including social effects, can be greater than the phenotypic variation (Bijma et al. 2007b). The impacts of these alternative selection methods on social interactions were easily observed from results of individual versus group selected chickens of (Muir 1996; Muir and Craig 1998). We observed that the group selected birds exhibited near perfect plumage and low or no mortality. In contrast, individually selected birds had decreased annual production, extensive feather pecking, and an increased mortality due to cannibalism and feather pecking. Clearly, the genetic correlation between individual and group productivity was negative yet group selection brought about a positive change.

Although individual selection has been the predominant method of domestication of poultry and livestock, when social interactions are present, individual selection is suboptimal and can be detrimental. In extreme cases, individual selection can increase allele frequency of so called Trojan genes. Trojan genes have conflicting impacts on alternative components of individual fitness, usually reproductive and viability fitness. Depending on the relative strength of selection on the two components, the improved reproductive fitness can increase frequency of the aberrant allele, whereas the reduced viability fitness of offspring may drive the population to eventual extinction. Trojan genes have been documented to occur naturally due to new mutations (Dawson 1969) and (Martin et al. 2002), and has been hypothesized as a potential risk for genetically engineered organisms (Muir and Howard 1999; Hedrick 2001; Howard et al. 2004). One possible mechanism for a population to overcome a Trojan gene would be classical group selection, where some groups did not possess the aberrant allele. However, there would be a strict necessity for no migration to occur among groups and complete extinction of affected groups.

The ultimate goal of domestication programs is sustainability. However, “classic” group selection, as defined by (Williams 1966), is not sustainable for the very reason it works, that is, entire families must be selected. As a result, the effective population size is greatly reduced, resulting in greater rates of inbreeding, which limits future gains due to random loss of favorable alleles (Robertson 1960; Hill 1985), and causes inbreeding depression (Hill and Robertson 1968; Lynch and Walsh 1998). In natural settings, a population may be large enough such that even with classic group selection, inbreeding occurs at a low enough rate to allow selection to dominate over drift, and new mutations to accumulate and the population to continue to evolve. However, genomic data have shown that much of the allelic diversity present in wide ancestors of poultry is not present in current commercial poultry operations (Muir et al. 2008), indicating that commercial populations have already small effective size, which would be further reduced with classic group selection. Thus, while classic group selection can result in rapid short-term responses, long-term breeding alternatives are needed that do not compromise effective population size, while still addressing negative social interactions. A possible alternative is multilevel selection, that is, shift the unit of selection back to the individual, but rear individuals in multiple small groups of related individuals, as with classical groups selection, rather than one large group. In this way the covariance within group will contribute to between-group differences and to the selection objective, thereby achieving some of the social benefits of group selection while at the same time increasing the number of families that contribute to the next generation.

Multilevel selection is a direct analogy of classical index selection based on the noninteraction model derived by Lush (Lush 1947a,b). For the social interaction case, the extension of the index to include interacting individuals was defined by Griffing (1977), as follows, let 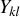 be the phenotype of the *l*th individual in the *k*th group (or family), then, the between and within group deviations can be combined in an index 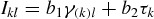, where 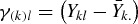 and 
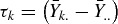
 are, respectively, the within- and between-group deviations, and *b*_1_, *b*_2_ are weights. Full between-group selection occurs when *b*_1_ = 0, individual selection results when *b*_1_ = *b*_2_, and multilevel selection results from other combinations. This index unified the concepts of multilevel, group, and within-group selection. Bijma and associates (2007a,b; Bijma and Wade 2008) independently derived similar results, but used only one parameter, “g” to define the strength of multilevel selection. They defined an index within the context of a group. For the *k*th group, the selection criterion can be represented as a combination of the contributions of the individual and its associates:


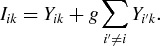


For *g* = 0, the criterion is the phenotype of the individual, and when *g* = 1, the criterion is the group performance. The relationship between weights in the two indices is


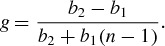


Breeders typically use selection programs where animals are ranked at the population level but reared in multiple small groups, such as with swine breeding. In such cases, animals are usually assigned to pens at random. However, as shown below, substantial gains in selection response may be possible simply by housing animals in kin groups as opposed to random, even though the model for estimating breeding values (EBVs) is the same.

Here we compare for the first time under controlled experimental settings, multilevel trait-based selection in kin versus random groups. We used Japanese quail (*Coturnix japonica*) as a model for natural populations that consist of many small groups or domesticated species of animals that are reared in pens and phenotypes measured on individuals, such as with swine (*Sus scrofa*), poultry broilers (*G. gallus domesticus*), and many aquacultured species. Japanese quail are known to exhibit strong competitive social interactions, including aggression and cannibalism (Mills et al. 1997; Huss et al. 2008).

## Methods and Materials

The experimental design was similar to that described by Muir (2005) who gave results using birds selected using an optimal index of the direct and IGEs and another with birds selected only on direct breeding values. In brief, the experimental consisted of a randomly bred line of quail kindly supplied by Dr. Henry Marks (USDA/ARS, Athens, Georgia). All experiments were conducted at the Purdue Poultry Research Center under animal care protocols approved by the Purdue University animal care committee. Injured birds were humanely euthanized. None of the birds were beak trimmed at any age.

### EXPERIMENTAL SETUP

Two experimental setups were compared: in each case the selection was based on classical Best Linear Unbiased Prediction (BLUP), but with alternative assignment of individuals to cages: (1) (Kin) by half-sib families and (2) (Random) a positive control. BLUP is a method of estimating breeding values (EBV) and is the same as an index that places optimal weights on within and between family deviations. If the families are reared in groups, as with assignment 1, selection based on BLUP EBV is multilevel because the family includes covariances with related individuals. If individuals are reared in random groups, as with setup 2, then the family include associations with random group members and is the same as individual selection.

Selection was for increased 43 day weight. All other factors, such as selection intensity, housing density, and feed, were kept constant. For each selection method, one set of cages was used for breeding and another for brooding and rearing. The breeder cages allowed individual single pair natural mating with automatic cup waters and trough feeders. The rooms were light tight, with automatic ventilation and lighting (14:10 h light:dark cycle maintained at full intensity and temperature was maintained at 27 ± 4 °C. The breeding program was maintained on an 85 day overlapping cycle.

The experimental designs for multilevel selection in groups composed of Kin and Random individuals were as follows: Starting at day of hatching, chicks were toe clipped to designate dam, and placed by sire family in brooding cages located in a room maintained at 38 ± 1 °C and fed ad lib a starter diet. At 14 days chicks were wing banded and moved to grow out cages. Grow out occurred in 24 cages (61 cm × 61 cm) with trough feeders 15.2 cm in length along the front of the cages. Water was provided by automatic drip nipples. For Kin groups, birds were moved as a sire family group, any extras were randomly eliminated, such that the number housed was a constant 16 per cage. For Random groups, birds were allocated to cages using stratified randomization, that is, no two birds from the same sire family were placed in the same cage, but otherwise at random. Birds were fed 240 g of mash once per day, this amount was adequate to meet all nutritional requirements provided the birds did not waste feed. At 43 days of age birds were sexed and weighed. Birds that died prior to weighing were usually the result of cannibalism, fighting, pecking, or other negative social interactions and were assigned a weight of zero to reflect the negative impacts of IGEs from cage associates. Breeding values were estimated (EBV) as described in the following section and selection decisions based on ranked EBVs. The birds with the highest EBV were used to replace breeders with lower EBVs in the breeder cages and allowed to mate. One male was mated to four females by daily rotation among cages. Eggs were collected daily starting 4 days later and continued for 2 weeks. Collected eggs were held in a cold room at 4°C to preserve the egg and prevent embryo development prior to setting. The eggs were then transferred to a commercial incubator with hatching 3 weeks later. The generation interval from hatch to hatch was 12 weeks. The process of mating and egg collection required 3 weeks, thus eggs for the next hatch were collected every 3 weeks. The first seven hatches were from the same parents because selection candidates were not fully mature and ready for mating until hatch 8. As such, results for the first seven hatches were combined to form the first mini generation (MG), and others were renumbered sequentially from the seventh hatch, that is, hatch 8 was the first hatch using selected parents and designated MG 1. For clarity, hatch 8 (MG 1) was produced from parents selected among the offspring of hatch 1; hatch 9 (MG 2) was produced from parents selected among the offspring of hatches 1 and 2, this process was repeated in this manner for 17 MG of selection. Breeders were fed ad lib a standard layer diet.

### MULTILEVEL SELECTION

Selection decisions were made based on ranked EBVs, which are an estimate of the expected genetic contribution of an individual to the offspring. We estimated breeding values, by BLUP (Henderson 1975; Henderson 1984; Henderson and Quaas 1976) using custom programs, based on the model


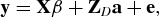


where **y** is a vector of observations, **β** a vector of fixed effects including the mean and sex, **a** a vector of ordinary (i.e., direct) breeding values, **e** a vector of residuals, and **X** and **Z**_D_ are incidence vectors linking observations to the causal variables. BLUP produces an estimate of **a**, the so-called EBVs.

Traditionally the use of BLUP increases the accuracy of EBVs by utilizing information from relatives. However, if relatives are in the same group, then BLUP also weighs the group performance, and selection on EBVs is thus multilevel selection (*g* > 0). In contrast, if relatives are in different groups, then the selection criterion does not include any weight on group performance (*g* = 0).

Quail show strong social interactions, creating IGEs on 43-day weight (see Table 1 in Results). Hence, 43-day weight results from both direct and IGEs,


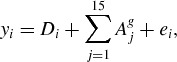


where 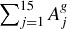 denotes the sum of the IGEs of the 15 associates in the same group as the focal individual. When relatives are in different groups, the selection criterion does not include any weight on group performance (*g* = 0), but the IGEs on the trait cause the EBV of the focal individual to depend on genes of its associates. Thus there is no multilevel selection on trait value (*g* = 0), but the selection criterion nevertheless depends on genes in associates. When relatives are in the same group, the selection criterion depends on genes in associates both due to IGEs on trait value, and due to multilevel selection on trait value (*g* > 0).

**Table 1:** Adjusted genetic parameters for quail selection experiment

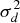 genetic variance of direct effect	24.60
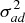 genetic covariance of direct and indirect genetic effect	−1.2
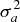 genetic variance of indirect genetic effects	1.04
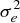 residual variance	124.5
ρ = Correlation among residuals	0.09

Expected responses were determined based on the theoretical expectations given by Bijma (2011) and Bijma and Wade (2008) as 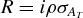, where


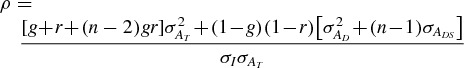


and 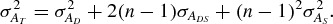


The *i* is the so-called intensity of selection, which follows from the selected proportion (Falconer and Mackay 1996), *ρ* is the accuracy of selection, which is the correlation between the selection criterion and the true breeding value, and 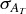 is the total heritable standard deviation in trait value, including both direct and IGEs (Bijma et al. 2007b; Bijma 2011).

To estimate genetic parameters required to calculate those predictions, Muir and colleagues (Muir and Schinckel 2002; Muir 2005) recast Griffing's model in terms of a mixed model methodology, with two random effects, one for the direct effect of the allele on the phenotype and another for IGEs on associates. The mixed model used was


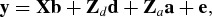


where ‘**b’** denotes a vector of fixed correction factors for the overall mean and sex effect, “**d**,” and “**a**” are vectors, respectively, of direct genetic effects and IGEs and **X**, Z*_d_*, and Z*_a_* are incidence matrices connecting observations to explanatory variables. The mixed model was further modified by Arango et al. (2005) who added a random effect to accont for a correlated residual due to the shared common environmental effect of the group. Bijma et al. (2007a) showed that the correlated residual is due to indirect environmental effects of one individual on another, similar to IGEs but due to nongenetic effects. Bijma et al. (2007a) also showed that if the correlated residuals are not properly accounted for, they upwardly bias the estimates of genetic parameters. We assumed the biases were of similar magnitude as those given by Bijma et al. (2007a) and adjusted the parameters given by Muir (2005) accordingly.

## Results and Discussion

**Figure 1:**
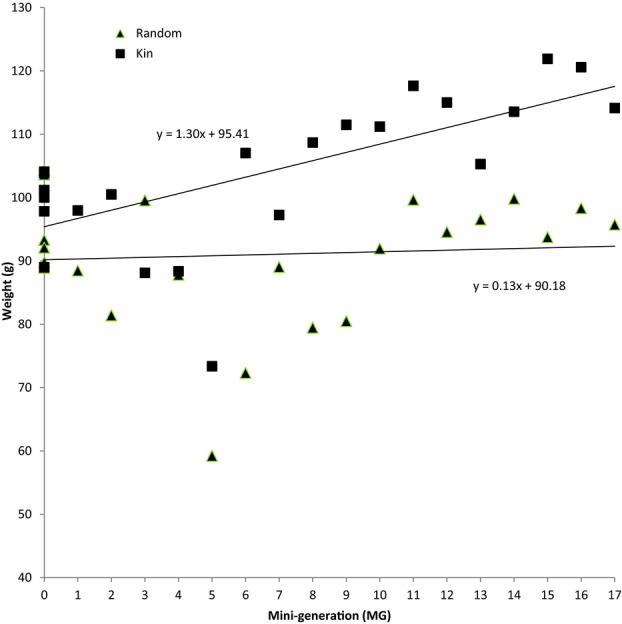
Quail body weight at 43 days of age by mini generation (MG) and selection program (Kin vs. Random).

**Figure 2:**
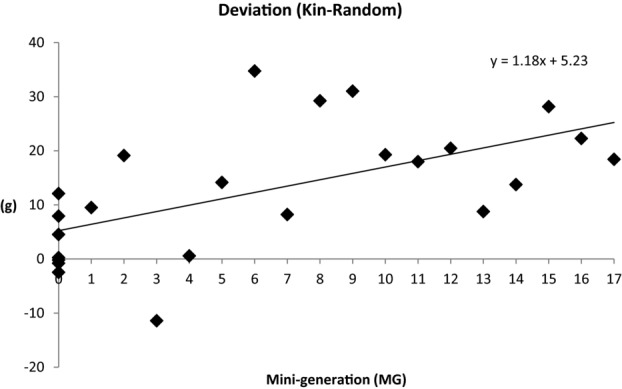
Deviations in weight between Kin versus Random selection.

As mentioned previously, multilevel selection results from an index in which the individual and others in the same group are weighted. The use of BLUP to estimate breeding values was approximately the same as using an index with weight of *g* = .23 for the summed performance of associates when individuals are housed as kin groups (see Appendix), and *g* = 0 when individuals are housed randomly. The strength of selection is measured relative to group selection, full group selection would have a strength of 1. The response to selection is also dependent on selection intensity (*i*) calculated as follows: The average selection intensity over males and females was approximately *i* = 1.15/generation and with five MG per generation, *i* = .23/MG). The observed responses to selection for 43-day weight for the 18 MG of selection are shown in Figure 1, and the estimated regression coefficients were *b* = 1.30 ± 0.31 g/MG for Kin and *b* = 0.13 ± 0.40 g/MG for Random. The difference is highly significant (*P* < 0.01) showing that response with Kin was an order of magnitude greater than with Random. The large drop in weights in MG 6 was found to be due to a bad batch of vitamin premix in the feed. Following replacement with new vitamin premix weights returned to normal. Analyzing deviations between weights of Kin versus Random (Fig. 2) removed the common environmental effects due to diet and showed that the differences in response was linear (*P* < 0.002), lack of fit was tested by adding a quadratic effect, which was found to be conservatively nonsignificant (*P* > 0.10).

**Figure 3:**
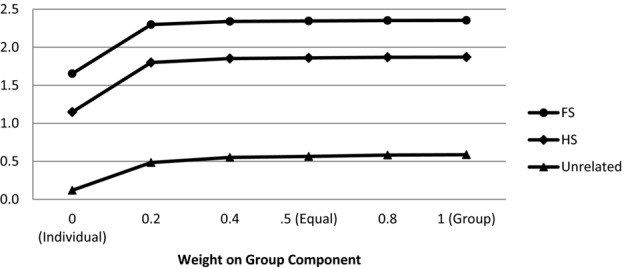
Expected response to selection with groups composed of unrelated, full sib, and half sibs.

Genetic variances and covariances for the base population were given by Muir (2005). These estimates were adjusted for bias, due to correlated residuals (Bijma et al. 2007a), and are given in Table 1. The expected responses for any strength (*g*) of multilevel selection are given in Figure 3. Specifically for *g* = .23 and *g* = 0, the expected response per MG with half sibs and unrelated groups is, respectively, 1.8 g/MG and 0.1 g/MG, which is greater than observed for Kin selection (1.30 ± 0.31), but slightly less than observed for Random (0.13 ± 0.40), however neither were significantly (*P* > 0.05) different from expectation.

**Figure 4:**
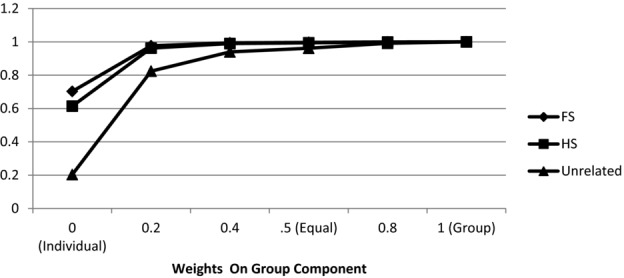
Response relative to groups composed of unrelated, full sib, and half sibs.

From Figure 3, for this set of parameters, and for the same group compositions, group selection is always expected to be superior to any other combination of multilevel selection, the worst case resulting from individual selection. When responses are compared as a proportion of that achievable with group selection (Fig. 4), if groups are composed of at least half sibs, near equivalent advance can be attained with even minor weight (*g* = .2) on the group component. In addition, even with unrelated groups, a minor weight on the group component (*g* = .2) results in the majority of the group response to be realized. But to achieve near equivalent group response, most of the weight (.8) must be placed on the group component. This result arises because relatedness and multilevel selection increase the utilization of heritable variation (Bijma 2011), and both factors enforce each other, as shows by the (*n* − 2)*gr* term in the expression for response given above.

Overall mortality due to fighting and cannibalism in MG 10–18 for Kin and Random grouping was, respectively, 6.6% and 8.5%, the difference, as tested by chi-square, was highly significant (*P* < 0.0002). Thus multilevel selection in kin groups was effective in reducing detrimental social interactions, which contributed to improved weight gain. The results observed in these experiments are similar to those we found with poultry layers (Craig and Muir 1996; Muir 1996) where group selection was able to overcome opposing effects of individual within group selection. An interesting theoretical result given by Bijma et al. (2007a) is that the total heritable variance can exceed the observed phenotypic variance indicating that the response to selection can be greater than the selection differential, and explains the results we observed with group selection in poultry layers where the realized heritability in the initial generations was >1. Basically the response to selection includes both the direct and the IGS's of the heritable social environment, not just the direct effects.

In terms of a sustainability of domestication breeding programs, simple multilevel selection is easy to implement and is expected to improve both productivity and animal wellbeing similar to group selection, but with somewhat lower levels of inbreeding because families are not the unit of selection as with group selection. These results were demonstrated in this experiment where a low value of **g** occurred, indicating that multilevel selection was primarily acting on individuals, yet was able to achieve near optimal response only attainable with between group selection. Alternatively, the direct and IGEs could be directly estimated for each individual using the methods given by Muir and associates (Muir and Schinckel 2002; Muir 2005; Bijma et al. 2007a) and select for total breeding value (TBV) using an index. Results from using that method were reported earlier (Muir and Schinckel 2002; Muir 2005) in a companion to this experiment. Selection on TBV increased 43-day weight by 0.52 ± 0.25 g/MG, which was significantly less than multilevel selection in kin groups reported here, but significantly greater than individual selection in random groups.

In addition to the reduced response of the two component approach, in practice reliable estimates of genetic parameters for both direct and indirect effects, as well as a random effect for a correlated residual, may be difficult to attain, including problems with estimability and convergence (Arango et al. 2005; Cantet and Cappa 2008), making construction of an optimal index problematic. Moreover, the use of an optimal index requires recording of individual phenotypes within group, which may be difficult for, for example, egg production. Nevertheless, the optimal breeding program, even with the TBV approach, is to rear animals in kin groups, in this way accuracy of selection for TBV is maximized (Ellen et al. 2007).

One objective of this research was to make the results relevant to breeders who usually use a single random effect mixed model to estimate breeding values, in which case the only procedural change the breeder would make is how animals are housed, thus making the transition to multilevel selection simple and easy to implement. Another focus was on how animal breeding experiments can demonstrate to evolutionary biologists that simple models of multilevel selection can accurately predict response to selection in real biological systems.

## Appendix: Strength (g) of Multilevel Selection

In the following, *n* is group size, *r* is relatedness. Genetic parameters are as define in Table 1. All sibs are kept in the same group as the candidate of selection. We approximated BLUP with an index of own performance and sib information. The weights of the selection index were derived based only on direct effects.

Let I be the selection index with weights *b_1_* and *b_2_*, respectively, on the individual and on sib information

Because the strength of selection is defined relative to group selection whose weights would be 1 for the individual and (n − 1) for the sibs, the strength of multilevel selection was *g* = 3.5/15 = .23.
